# ARP2/3 localization in Arabidopsis leaf pavement cells: a diversity of intracellular pools and cytoskeletal interactions

**DOI:** 10.3389/fpls.2013.00238

**Published:** 2013-07-12

**Authors:** Chunhua Zhang, Eileen L. Mallery, Daniel B. Szymanski

**Affiliations:** ^1^Department of Agronomy, Purdue UniversityWest Lafayette, IN, USA; ^2^Department of Biology, Purdue UniversityWest Lafayette, IN, USA

**Keywords:** ARP2/3, actin, microtubules, leaf pavement cells, localization

## Abstract

In plant cells the actin cytoskeleton adopts many configurations, but is best understood as an unstable, interconnected track that rearranges to define the patterns of long distance transport of organelles during growth. Actin filaments do not form spontaneously; instead filament nucleators, such as the evolutionarily conserved actin-related protein (ARP) 2/3 complex, can efficiently generate new actin filament networks when in a fully activated state. A growing number of genetic experiments have shown that ARP2/3 is necessary for morphogenesis in processes that range from tip growth during root nodule formation to the diffuse polarized growth of leaf trichomes and pavement cells. Although progress has been rapid in the identification of proteins that function in series to positively regulate ARP2/3, less has been learned about the actual function of ARP2/3 in cells. In this paper, we analyze the localization of ARP2/3 in Arabidopsis leaf pavement cells. We detect a pool of ARP2/3 in the nucleus, and also find that ARP2/3 is efficiently and specifically clustered on multiple organelle surfaces and associates with both the actin filament and microtubule cytoskeletons. Our mutant analyses and ARP2/3 and actin double labeling experiments indicate that the clustering of ARP2/3 on organelle surfaces and an association with actin bundles does not necessarily reflect an active pool of ARP2/3, and instead most of the complex appears to exist as a latent organelle-associated pool.

## Introduction

In plant cells, the actin cytoskeleton is a dynamic architectural control element that functions at spatial scales of the cell to anchor organelles at specific locations and serves as a track that defines the cellular patterns of organelle motility that supply the growth process. Although potential similarities among the boundary conditions of crawling animal cells with a retractable leading edge and growing plant cells have been discussed (Mathur, 2005), the mechanics of cell shape change in plants is completely different. Complex interactions between turgor-pressure-driven cell wall tension and cell wall anisotropy define the specific geometries of cell shape change (Baskin, 2005; Szymanski and Cosgrove, 2009). One function of the actin cytoskeleton is to control the secretion patterns of signaling proteins, wall-modifying enzymes, and polysaccharide cargo that polarize the cytoplasm and determine the physical properties of the wall texture (Geldner et al., 2001; Bosch and Hepler, 2005; Leucci et al., 2007; Gutierrez et al., 2009). Plant cells, unlike those of their crawling mammalian counterparts, do not have actin networks that obviously reflect cell shape. Instead the actin system is dominated by instabilities, from the rapid turnover of individual actin filaments to the constant rearrangement of the linkages within the actin bundle network (Staiger et al., 2009; Szymanski and Cosgrove, 2009; Smertenko et al., 2010). A major challenge in the morphogenesis field is to better understand the functional importance of specific subsets of actin arrays and the cellular control of their assembly and turnover (Szymanski, 2005; Staiger and Blanchoin, 2006; Prigge and Bezanilla, 2010).

The actin-related protein (ARP)2/3 complex is one known actin filament nucleator, whose composition (Mathur et al., 2003b; El-Assal et al., 2004b) and assembly (Kotchoni et al., 2009) are conserved among yeasts, metazoans and plants. ARP2/3 is intrinsically inactive (Higgs et al., 1999), but is converted into a potent actin filament nucleator by a diverse class of proteins termed nucleation promoting factors (Welch and Mullins, 2002; Campellone and Welch, 2010). Plants are unique because they rely on a single family of ARP2/3 activators termed SCAR/WAVE (Zhang et al., 2008). Arabidopsis SCARs function in the context of another heteromeric complex termed the WAVE/SCAR-regulatory complex (W/SRC) (Deeks et al., 2004; El-Assal et al., 2004a; Basu et al., 2005; Zhang et al., 2005; Djakovic et al., 2006; Le et al., 2006; Jorgens et al., 2010). W/SRC appears to convert activating ROP-GTP signals from the DOCK-family-GEF SPIKE1 (Basu et al., 2008) into an ARP2/3 activation response. However, the mechanisms of W/SRC and ARP2/3 are complex (Stradal and Scita, 2006; Lebensohn and Kirschner, 2009; Chen et al., 2010), and even in the best-developed experimental systems, the cellular control of ARP2/3 activation is not well-understood (Rottner et al., 2010; Rotty et al., 2013).

The W/SRC-ARP2/3 growth control pathway is widely deployed in plants during tip growth (Harries et al., 2005; Perroud and Quatrano, 2006), root nodule formation (Yokota et al., 2009; Miyahara et al., 2010), and epidermal morphogenesis in trichomes, ordinary cylindrical epidermal cells, and interdigitated pavement cells (Le et al., 2003; Mathur et al., 2003b; Djakovic et al., 2006; Le et al., 2006). There are clear actin bundle positioning defects in *arp2/3* trichomes (Szymanski et al., 1999; Le et al., 2003) and cylindrical epidermal cells in the root and shoot (Dyachok et al., 2011), and in each case a disorganized longitudinal actin bundle network is correlated with defective cell elongation. In pavement cells, the actin defects in *arp2/3* are subtle, and in general both the function of actin in this cell type and the morphogenesis process itself are not well-characterized. For example, the timing and location of polarized growth during pavement cell morphogenesis are not known. In cotyledon pavement cells, lobe formation is discontinuous, and alternates between phases of cell morphogenesis that include symmetry breaking and lobe formation and extended growth phases, on the order of days, during which cell size increases, but the cell shape is more or less maintained (Zhang et al., 2011). Therefore, in cotyledons and also in leaves (Elsner et al., 2012) it is not possible to deduce anything about the instantaneous growth behavior of the cell from a single time point image of a cell. Although a local enrichment of “fine F-actin” in the subsets of previously existing lobes has been reported in some cases (Fu et al., 2002, 2005; Li et al., 2003; Xu et al., 2010), it is unclear if these actin-enriched sub-regions of the cell reflect lobe initiation events or polarized growth. In contrast, several other live-cell imaging and immunolocalization studies on the same cell type failed to detect or report a correlation between the existence of cell lobes and the presence of dense actin meshworks (Qiu et al., 2002; Mathur et al., 2003a; Djakovic et al., 2006). The reasons for these discrepancies are unclear, but at present, the relationships among ARP2/3, actin, and pavement cell shape control are unknown.

Localization data on ARP2/3, actin, and its positive regulators provide useful clues about the mechanisms of its cellular deployment. In non-plant systems, the compartmentalization of ARP2/3 activity can occur at the micron-scale of regulated nuclear import (Goley et al., 2006; Weston et al., 2012) to the nanoscale, in which the protein complexes are sequentially recruited to organelle subdomains to distort and/or fuse membranes (Svitkina and Borisy, 1999; Eitzen et al., 2002; Kaksonen et al., 2003; Chhabra and Higgs, 2007; Derivery et al., 2009; Gomez and Billadeau, 2009; Carnell et al., 2011; Harbour et al., 2011). In plants, useful data on the localization of ARP2/3 and its activators are accumulating. SPK1 is detected in the nucleus, as are the W/SRC subunits NAP1 and SCAR1 (Zhang et al., 2010, 2013; Dyachok et al., 2011). The W/SRC subunits BRK1 and SCAR form a sub-complex, and depending on the cell type, may define active pools of ARP2/3 on intracellular organelles or at the plasma membrane (Frank et al., 2004; Basu et al., 2005; Zhang et al., 2005, 2008; Djakovic et al., 2006; Le et al., 2006; Perroud and Quatrano, 2008).

In thick walled plant and algal cells, ARP2/3 appears to be associated with diffuse actin meshworks, actin bundles and organelles (Van Gestel et al., 2003; Maisch et al., 2009), and in some cases, a polarized localization is correlated with asymmetric growth (Hable and Kropf, 2005; Perroud and Quatrano, 2006). In tobacco BY-2 cells that employ a diffuse or intercalary growth mechanism, ARP2/3 is reported to colocalize with and generate actin bundles (Maisch et al., 2009). However, ARP2/3 is tightly associated with organelles (Perroud and Quatrano, 2006, 2008; Kotchoni et al., 2009), and bundle-associated ARP2/3 could reflect acto-myosin-dependent transport of ARP2/3 that is clustered on an organelle surface. In Arabidopsis leaf pavement cells, BRK1:YFP and GFP:SCAR1 localize to the plasma membrane and to punctae of unknown identity that often reside at 3-way cell wall junctions (Dyachok et al., 2008, 2011). At present there is a strong need to determine the localization of ARP2/3 in pavement cells.

In this paper we use a fully functional epitope-tagged version of the ARPC4 subunit of ARP2/3 to clearly define at least five different intracellular pools of the complex. Our pavement cell data are consistent with a model in which most of the ARP2/3 in the cell is inactive, and its concentration in the nucleus and its clustering on organelle surfaces requires neither a fully assembled W/SRC nor full ARP2/3 activation. In the shoot, ARP2/3 coats the surface of biochemically and morphologically distinct organelles that are efficiently coupled to the actin filament and microtubule cytoskeletons.

## Results

**Figure 1:**
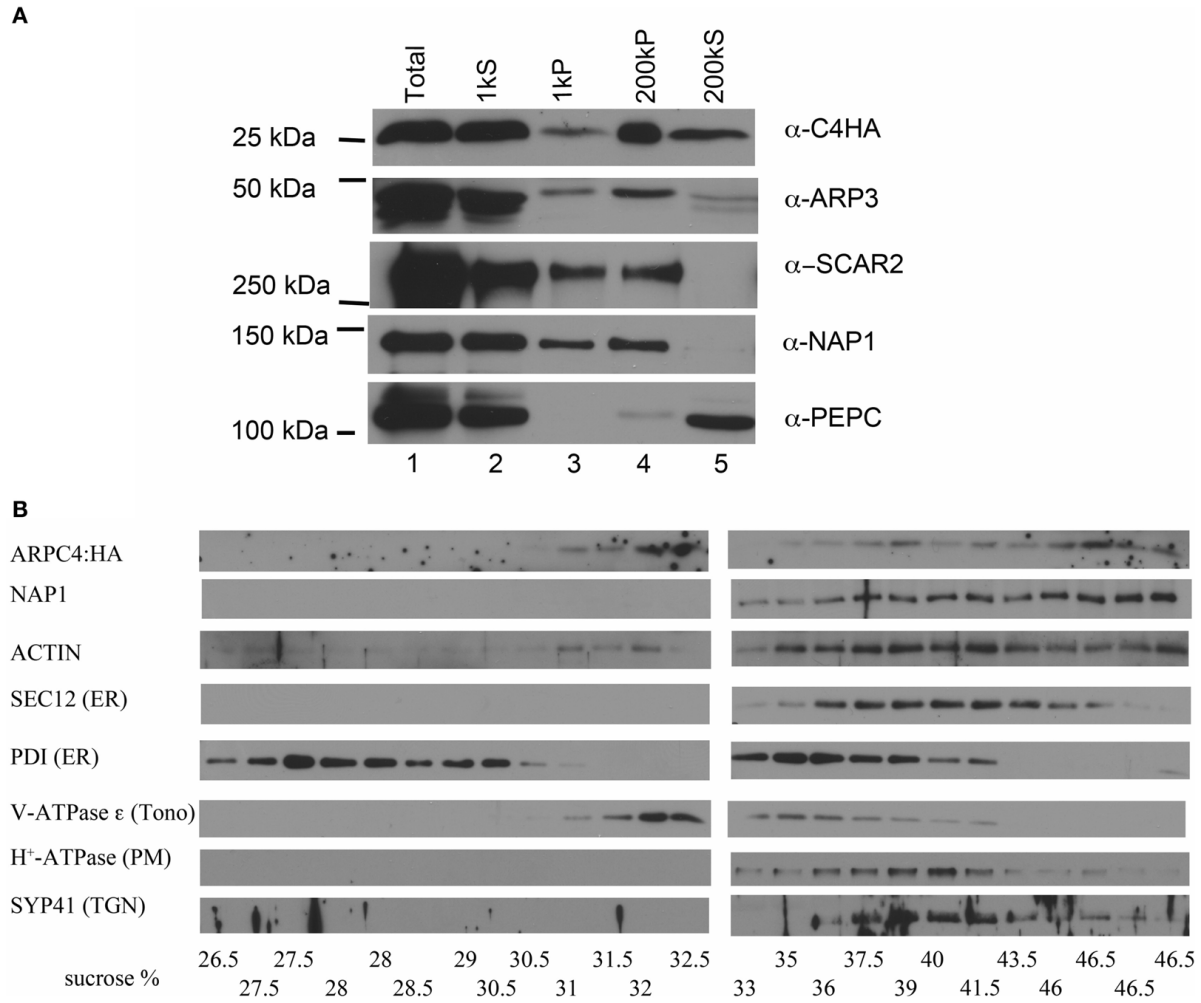
**ARP2/3 specifically associates with distinct organelles. (A)** Differential centrifugation of leaf cytosol and membrane fractions. Total extract was sequentially centrifuged at 1000 and 200,000 g. Equal cellular proportions of each fraction were separated on SDS-PAGE gels and probed with antibodies as indicated in the figure. **(B)** Sucrose velocity gradient fractions of crude microsomes probed in western blots with antibodies to ARPC4:HA, NAP1, ACTIN, and marker antibodies to proteins with a known organelle localization. PDI, protein disulfide isomerase (ER lumen), SEC12 (ER), vATPase ε, (tonoplast), H^+^-ATPase (plasma membrane), SYP41, trans-Golgi Network (TGN). The measured sucrose concentrations of each fraction are listed along the bottom of the figure.

We had previously reported that Arabidopsis ARPC4:HA is functional in trichomes, exists solely within an ARP2/3 complex, and is strongly associated with microsomes (Kotchoni et al., 2009). Consistent with our previously published results (Kotchoni et al., 2009), we found that ARP2/3 associates strongly with cell membranes (Figure 1). We extended this result, and found that the W/SRC subunits NAP1 and SCAR2 also partition strongly into the crude microsomal factions (Figure 1A, lane 4). The ARP2/3 complex, detected with the ARPC4:HA and the endogenous ARP3 subunit, is present in the low speed pellet fraction that contains large organelles such as nuclei, mitochondria, and chloroplasts. NAP1 and SCAR2 are also detected in the low speed pellet fraction (Figure 1A, lane 3). This signal is not due to the presence of unbroken cells, because the cytosol marker phosphoenol-pyruvate carboxylase (PEPC) is not detected in this fraction. We consistently detected a pool of soluble ARP2/3 (Figure 1A, lane 5); however, soluble NAP1 and SCAR2 are present in only trace amounts (Zhang et al., 2013).

To determine if there was any specificity to the interaction of ARP2/3 with membranes, we separated crude microsomes on continuous sucrose velocity gradients. Interestingly ARPC4:HA consistently had a bimodal distribution with peaks centered on 32 and 46.5% sucrose, indicating that ARP2/3 associated with distinct organelle membranes. We attempted to confirm these results with the anti-ARP3 antibody, but ARP3 was more sensitive to proteolysis compared to ARPC4:HA, and in three biological replicates, ARP3 had peak signal centered on 32 or 46% sucrose but was not consistently detected. Proteolysis of ARP2 has also been reported in a brown algae (Hable and Kropf, 2005). The results with ARPC4:HA indicated that ARP2/3 specifically associates with membranes, because it did not bleed across the entire gradient as would be expected for a protein that interacted non-specifically with membranes. Interestingly, the known W/SRC complex subunit NAP1 overlapped significantly with ARP2/3 in the higher density fractions, but did not have a corresponding peak centered on 32% sucrose (Figure 1B). We tested the distribution of ACTIN and it also was present in the most dense fraction, but was distributed more broadly compared to ARP2/3 and NAP1 (Figure 1B).

We have shown that NAP1 is concentrated on the ER surface (Zhang et al., 2013). To get an indication of the possible identity of the organelles that accumulate ARP2/3, the same sucrose density fractions were probed with a panel of marker antibodies to known compartments. SPK1 accumulates within specialized domains of the ER that are marked by several ER exit site markers and the SAR1 GEF SEC12 (Zhang et al., 2010). We find that the pool of ARP2/3 and NAP1 in the dense fraction partially overlapped with SEC12. Surprisingly, another ER marker, protein disulfide isomerase (PDI), has a bimodal distribution with peaks at 28% and 36% sucrose that did not co-purify with SEC12, ARP2/3, and NAP1. The dense ARP2/3-positive vesicles were resolved from the tonoplast marker V-ATPase-ε; however, ARP2/3 signal overlapped significantly with trans-Golgi network and plasma membrane markers. Clearly this technique is not sufficient to determine the exact identity of the ARP2/3-positive organelles.

### ARP2/3 localization in pavement cells

**Figure 2:**
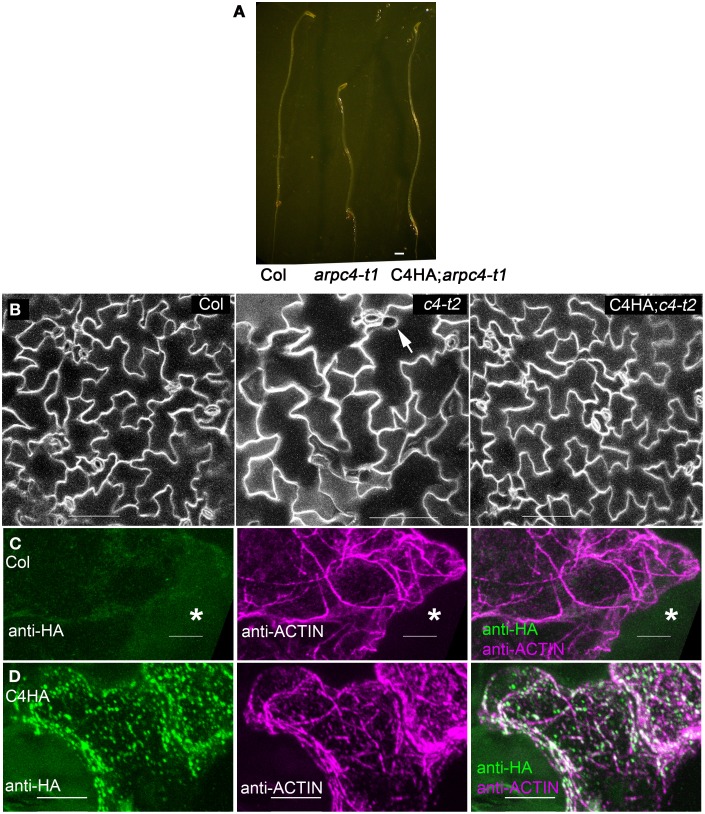
**ARPC4:HA is detectable in leaf pavement cells. (A)** ARPC4:HA restores etiolated hypocotyl elongation defect of *arpc4.* Seven days old dark-grown wildtype (left), *arpc4* (middle) and *arpc4*;ARPC4:HA (right) seedlings. **(B)** ARPC4:HA restores cotyledon pavement cell expansion phenotype of *arpc4*. FM1-43 staining of 12 days after germination cotyledon pavement cells from wildtype (left), *arpc4* (middle) and *arpc4*;ARPC4:HA (right). The arrow in the middle panel indicates a gap between pavement cells in *arpc4*. **(C)** Maximum Z-projection of series of confocal images of wild-type (Col) pavement cell labeled with anti-HA and anti-ACTIN antibodies. ^*^labels a cell that was not cracked during freeze shattering in liquid nitrogen and was not labeled for either actin or ARPC4:HA. **(D)** Maximum Z-projection of series of confocal images of ARPC4HA transgenic line labeled with anti-HA and anti-ACTIN antibodies. Bars: 1 mm in **(A)** 100 μm in **(B)** 5 μm in **(C,D)**.

We next sought to gain a more refined description of the subcellular distribution of ARP2/3 in localization experiments. Prior to these analyses we needed to establish the ARPC4:HA is functional throughout the shoot. We found that ARPC4:HA transgene expression reversed well-known shoot phenotypes (Table 1) such as the reduced hypocotyl elongation in the dark (Figure 2A), the reduced complexity of pavement cells, and cell-cell adhesion defects in cotyledons (Figure 2B). Circularity is a dimensionless shape factor that has a value of 1 for a circle and decreasing values as cell shape complexity increases. In the cotyledon epidermis, ARPC4:HA had a circularity of 0.21, which was almost identical to the wild type and was significantly less than the value of 0.34 that was measured in a population of *arpc4* pavement cells. Cell-cell adhesion defects have been frequently reported in the leaf epidermis (Qiu et al., 2002; Le et al., 2003; Mathur et al., 2003b; Zhang et al., 2005; Djakovic et al., 2006) and are present in *arpc4* cotyledons (Table 1). The ARPC4:HA transgene also reversed that phenotype; however, one gap was detected in 11 image fields (each image field was in the size of 630 × 630 μm) of the ARPC4:HA *arpc4* line. These data demonstrate the functionality of ARPC4:HA in a variety of cell types and validates its use as a tool to localize the ARP2/3 complex in pavement cells.

**Table 1:** **ARPC4:HA is functional in pavement cells and in dark grown hypocotyls**.

**Parameters**	**Col**	***arpc4-t2***	**ARPC4:HA;*c4t2***
Hypocotyl length (mm)	19.5 ± 2.3 (33) a	13.6 ± 2.2 (31) b	18.0 ± 2.2 (32) a
Pavement cells			
Perimeter (μm)	864.3 ± 207.6 (54) a	679.4 ± 234.8 (55) b	1068.7 ± 484.5 (53) c
Area (μm^2^)	12677 ± 3575 (54) a	12147 ± 5624 (55) a	16605 ± 8891 (53) b
Circularity	0.22 ± 0.06 (54) a	0.34 ± 0.09 (55) b	0.21 ± 0.08 (53) a
Skeleton ends	12.2 ± 2.9 (54) a	8.9 ± 2.7 (55) b	13.3 ± 5.4 (53) a
Gaps/mm^2^	0 (13)	10.2 ± 15.1 (11)	0.2 ± 0.8 (17)

All parameters are shown as mean values ± StDev. The numbers in the parentheses indicate the sample's size. For hypocotyl length, it is shown as the numbers of seedlings measured. For cell perimeter, area, circularity, and skeleton ends it indicates the numbers of cells measured. For gap frequency quantification, it indicates the number of image fields. The letters indicate ANOVA comparison test groupings. Mean values with the same letter in the group are not significantly different (α = 0.95).

Freeze shattering is a robust method to localize the cytoskeleton and endomembrane systems in plant cells (Wasteneys et al., 1997; Qiu et al., 2002; Zhang et al., 2010). In localization experiments we found that ARPC4:HA was easily solubilized by detergents, and it was necessary to include an 3-Maleimidobenzoic acid *N*-hydroxysuccinimide ester (MBS) pre-fixation step to retain ARPC4:HA. Our top choice would be to localize ARP2/3 in blunt tipped stage 4 trichomes, where the distorted mutants first display strong actin and cell shape defects (Szymanski et al., 1999; Le et al., 2003). However, the establishment of reliable methods for ARPC4:HA immunolocalization was technically challenging, (see Methods) and despite the fact that crude microsomal ARP2/3 is resistant to extraction by 1% triton X-100 (Kotchoni et al., 2009), optimized fixation conditions were required to localize ARP2/3, and using these methods we could not reliably label ARP2/3 or actin in cells with a dense cytoplasm, like stage 4 trichomes.

Pavement cells become highly vacuolated at an early developmental stage and they were more efficiently double-labeled for ARPC4:HA and actin. However, in pavement cells, the relationship between the localization of W/SRC proteins to 3-way cell wall junctions (Dyachok et al., 2008), the arrangement of the cytoskleleton, and the less convoluted shape of the arp2/3 mutants (Djakovic et al., 2006; Le et al., 2006; Zhang et al., 2008) is not at all clear. The specific goal of this work is to determine the localization patterns of ARP2/3 in pavement cells, and to use W/SRC signaling mutants and actin double-labeling to experimentally identify the active pools of the complex. In control anti-HA immunolocalization experiments using untransformed wild-type plants, a very low level of background ARPC4:HA signal was similar to the background signal of unlabeled cells (Figure 2C). Identical labeling experiments conducted with the ARPC4:HA; *arpc4* lines revealed clear signal in fractured cells that were accessible to the antibody (Figure 2D).

**Figure 3:**
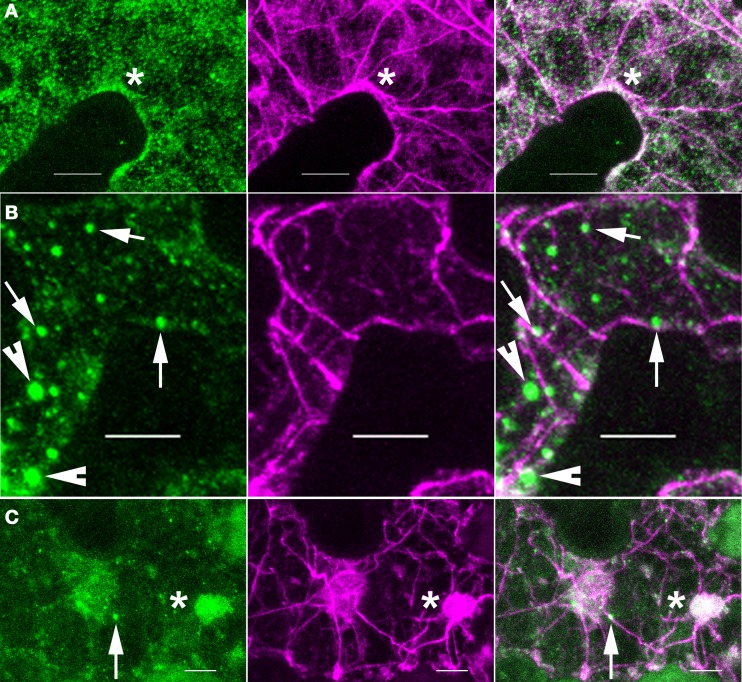
**ARP2/3 is concentrated on a heterogenous population of organelles, most of which are adjacent to actin bundles**. Left panels, anti-HA. Middle panels, anti-ACTIN. Right panels, merge of left and right panels. **(A)** ARP2/3 has a patchy granular appearance in some pavement cells. **(B)** ARP2/3 is localized to small punctate structures of approximately 1 μm in diameter. **(C)** ARP2/3 has a clustered distribution on the surface of large organelle that are greater than 2 μm. Bars, 5 μm. Arrows, ~0.5 μm size class of ARP2/3-positive organelles. Arrowheads, ~1 μm size of ARP2/3-positive organelles. ^*^region of ARP2/3 and actin colocalization.

The pattern of ARP2/3 localization was quite variable. About 72% (*N* = 53) of the double-labeled cells had a granular pattern of ARP2/3 signal that was always somewhat patchy (Figure 3A). The patchy ARP2/3 signal could originate from a soluble pool with a brightness that reflects the uneven thickness of the cytosol in vacuolated cells and/or a loose association of ARP2/3 with a membrane system. We cannot rule out the existence of a plasma-membrane-associated pool of ARP2/3. However, the extreme cell periphery was not a consistent source of strong ARP2/3 signal, and we did not detect abundant cortical punctae that resembled the distribution of endocytic sites (Konopka et al., 2008). Furthermore, the relatively mild phenotype of distorted mutants is not consistent with an essential function for ARP2/3 in endocytosis.

Many pavement cells also had a population of punctate ARP2/3 that was either spherical, slightly oblong, or cylindrical. Given the known membrane-association of ARP2/3 (Kotchoni et al., 2009) and the specific interactions of ARP2/3 with organelle surfaces (Figure 1B), the punctate ARP2/3 signal reflected the accumulation of ARP2/3 on specific organelles or subdomains within an organelle. The number of ARP2/3-positive organelles per field varied among cells and according to size, but they were always 2–6 times brighter than the more diffuse ARP2/3 signal. Using intensity-based thresholding, the maximum diameter of ARP2/3-postive organelles ranged from 0.4 to 3.7 μm with a mean 1.0 ± 0.5 μm (*N* = 168). The relatively large ARP2/3-positive organelles greater than 1 μm in diameter were relatively rare, with cells having either none or 4 or 5 at the most. The smaller punctae <0.5 μm in diameter tended to be more abundant with some cells having more than 50 such particles (Figures 2D, 3B). The largest size class greater than 2 μm (Figure 3C) was the most rare, with only four examples detected within the population of cells that were analyzed. Our fixation and localization methods accurately capture the sizes and shapes of organelles like the Golgi and ER bodies (Zhang et al., 2010, 2013); however, the effect of fixation on the morphology of ARP2/3-positive organelles is not known. Additional work using live cell imaging and high pressure freeze fixation and TEM will be needed to validate these results and understand how ARP2/3 might affect organelle shape and dynamics.

**Figure 4:**
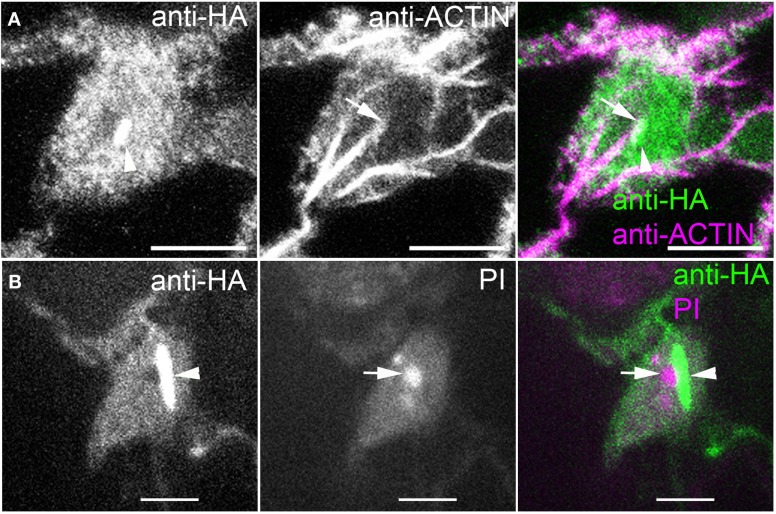
**ARP2/3 is localized to the nucleus and is concentrated within a rod-like structure that is adjacent to the nucleolus. (A)** Double labeling of ARPC4HA and ACTIN in the nucleus region of a pavement cell. Arrowhead, perinucleolar ARP2/3-positive rod, arrow, actin bundle that terminates at one end of the perinucleolar ARP2/3 rod. **(B)** Immunolabeling of ARPC4HA in the nucleus region of a pavement cell and the nucleus is stained by propidium iodide (PI). Arrow, nucleolus; Arrowhead, spindle shaped ARP2/3-positive rod; Bars, 2 μm in **(A)** and 5 μm in **(B)**.

We also consistently detected ARP2/3 inside a very large organelle that was suspected to be the nucleus based on its size, oval shape, number (one per cell), and position near the bottom of the pavement cells (Figure 4). This encapsulated ARP2/3 signal often had both a diffuse component and a striking, bright rod-like ARP2/3-positive structure that was nearly always adjacent to the nucleolus. The nucleolus was identified by staining anti-HA-labeled samples with propidium iodide (PI) to detect the abundant rRNAs (Figure 4B) and it was also visible in anti-HA labeled samples in negative contrast because ARP2/3 was apparently excluded from this subnuclear organelle. The perinucleolar ARP2/3 rod never strongly overlapped with actin bundles in the nucleus, but in projected images, it was common to detect thick actin bundles on the outer surface of the nucleus that terminated near one end of the perinucleolar ARP2/3 rod (Figure 4A). It is possible that undetectable individual filaments or some other linker molecules connect the perinucleolar rod with a nearby cytoplasmic actin bundle.

### Localization of active ARP2/3

In non-plant systems, it is common to define active ARP2/3 based on its colocalization with actin at maturing endosomes (Winter et al., 1999; Kaksonen et al., 2003) or in the periphery of lamellipodia (Svitkina and Borisy, 1999). In some instances heterologous antibodies that recognized proteins of approximately the correct size were used in localization experiments (Fiserova et al., 2006), but the specificity of these antibodies is very difficult to prove. For example, we found that an antibody against human ARP3 (Machesky et al., 1997) recognized a protein of the correct size in Arabidopsis; however, the signal was due to non-specific binding, because the same size protein was also detected in an *arp3*/*dis1-1* null line (Eileen L. Mallery, unpublished). In another report using tobacco BY-2 cells and antibodies against the tobacco complex, the ARP2/3 signal consists of bright punctae that are broadly distributed in the cell, but have an increased density in young cells (Maisch et al., 2009). A subset of the ARP2/3 punctae appeared to be physically touching actin bundles and occasionally were observed at the vertex of two intersecting bundles, prompting the authors to conclude that they were detecting active ARP2/3 that generated actin bundle/filament network (Maisch et al., 2009).

**Figure 5:**
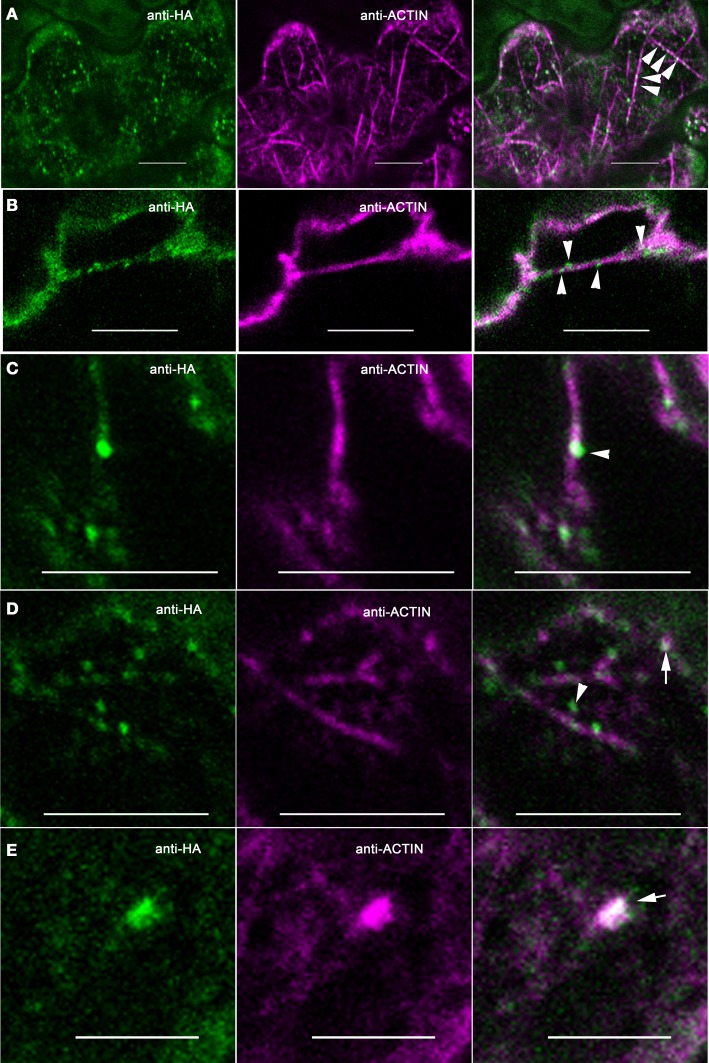
**ARP2/3 partially colocalizes with multiple actin networks in pavement cells**. Single plane confocal images from double-labeled pavement cells. **(A)** Some granular ARPC4 structures decorate along actin bundles, as indicated by the arrowheads in the right panel. **(B)**. Some granular ARPC4 structures are associated with actin bundles in the transvacuolar strands as indicated by the arrowheads. **(C)** Example of punctate ARPC4 adjacent to an actin bundle, as indicated by the arrowhead. **(D)** Examples of punctate ARPC4 that are independent of actin filaments or bundles as indicated by the arrowhead and the punctate ARPC4 that colocalizes with actin as indicated by the arrow. **(E)** Example of punctate ARPC4 that colocalizes with actin at some non-filamentous structure, as indicated by the arrow. Bars, 5 μm. Arrowhead, ARP2/3 adjacent to actin filaments or bundles, Arrows, regions of colocalization of ARP2/3 and actin.

In our analyses of the cytoplasmic pools of ARP2/3 we observed different types of colocalization with actin, most of which did not clearly identify active ARP2/3. For example, most of the ARP2/3-decorated organelles in the ~0.5 μm size class only partially overlapped with actin bundles and appeared to touch the bundle at a very localized region on the organelle surface (Figure 3B). The smaller size class of ARP2/3-positive organelles also decorated actin bundles and filaments (Figures 2D, 5A through 5D). The small punctae had no defined spacing along the bundles and their presence was largely independent of the presence of branched actin filaments, making it impossible to unequivocally identify active ARP2/3 at the vertex of branched actin filaments. Of the 112 ARP2/3-positive organelles that were manually screened in confocal image stacks for signal overlap with actin, 100 were adjacent to, and clearly touching actin bundles. This type of colocalization occurred in the cortical cytoplasm (Figures 5A,C,D) and within the cytosol of transvacuolar strands and sheets (Figure 5B); the latter transvacuolar space is less confined, and allows one to assess true colocalization. Transvacuolar strands and sheets are known locations for acto-myosin-dependent transport. The putatively soluble or weakly membrane-associated pool of ARP2/3 had a granular appearance, and qualitatively, the patchy regions of ARP2/3 signal tended to be near actin filament and bundle networks. As would be expected for a broad and diffuse signal, some of the ARP2/3 overlapped with the actin cytoskeleton (Figure 3A).

We performed a series of cross correlations analyses to test for an effect of ARP2/3 signal density on the extent of colocalization. For the 48 image slices from 15 independent confocal image stacks that were used for colocalization quantification, the overall cross correlations of the ARP2/3 and actin signals were moderate, with mean Pearson correlation coefficient (PCC) of 0.61 ± 0.17 (*N* = 48). The signals from each of the 48 ARP2/3 images were randomized in 10 simulated images and retested for colocalization with actin (Costes et al., 2004). None of the 480 simulated PCC values generated from randomized ARP2/3 signal and original actin images was higher than the PCC values from original, real images. The calculated probability of real colocalization existing between ARP2/3 and actin was 1.0, which indicates that colocalization we observed between ARP2/3 and actin was not due to chance events.

**Figure 6:**
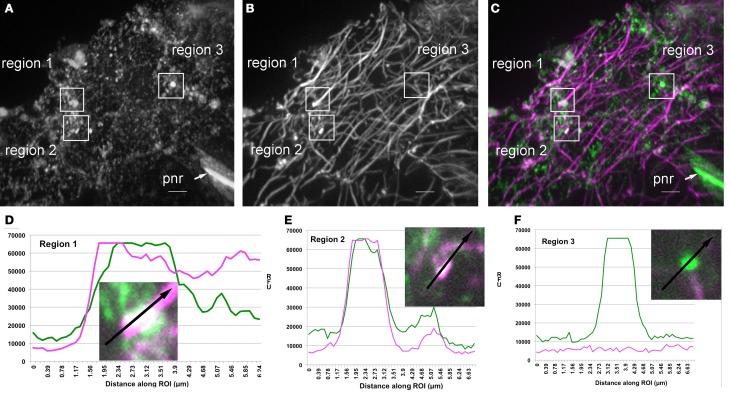
**Partial colocalization of ARP2/3 with microtubules and putative microtubule fragments in leaf pavement cells. (A–C)** Single channel and image overlay projections of confocal images of ARP2/3 and tubulin signal in a leaf pavement cell. For **(A–C)** a standard deviation projection method was used on 40 image planes to provide an overview of the regions that are highlighted in **(D–F)**. Colocalization analysis was performed exclusively within single image planes. **(A)** Anti-ARPC4:HA signal. **(B)** Anti-α-tubulin. **(C)** Overlay of ARPC4:HA (green) and tubulin (magenta) signals. Regions of interest that were analyzed for signal quantification in **(D–F)** are labeled on the figure. pnr, perinucleolar rod, bar = 5 μm. **(D)** Relative fluorescence intensity of ARP2/3 (green) and tubulin (magenta) signals along the indicated line within region 1. **(E)** Relative fluorescence intensity of ARP2/3 (green) and tubulin (magenta) signals along the indicated line within region 2. **(F)** Relative fluorescence intensity of ARP2/3 (green) and tubulin (magenta) signals along the indicated line within region 3, which is in the same image plane as region 2 that contains a large ARP2/3-positive organelle that is adjacent to an underlying microtubule. Insets, high magnification views of the regions labeled in **(C)**.

Genetic interactions between ARP2/3 and microtubules have been measured in developing trichomes (Zhang et al., 2005). Therefore, we compared the colocalization of ARP2/3 with actin and microtubules. An overlap of ARP2/3 and microtubule signal was apparent (Figure 6), and the PCC between the ARP2/3 and microtubules was 0.54 ± 0.15 (*N* = 48). None of the 488 PCC values obtained from randomized ARP2/3 signal was higher than the PCCs from the original images. Again, the calculated probability of true colocalization was 1.0, indicating that the colocalization between ARP2/3 and microtubues was not due to chance overlap. We also found that the PCC value between ARP2/3 and microtubules was not significantly different from that observed between ARP2/3 and actin (Students' *T*-test, *p* = 0.24, *N* = 48). These image quantification results indicate that the ARP2/3 colocalizes with both the actin and microtubule cytoskeletons.

A manual inspection of the images identified many instances in which bright ARP2/3 punctae were either touching or completely overlapped with anti-tubulin signal (Figure 6). For example, of 71 bright ARP2/3-positive structures >0.5 μm that were present in 37 different cells, 37% were adjacent to and in apparent physical contact with microtubules, 48% were at isolated organelles that had no overlap with the microtubule signal (although 5/26 had only a ~2 pixel separation from the microtubules), and 15% of the ARP2/3-postive structures had a more complete signal overlap with either microtubule ends (Figure 6D) or putative microtubule fragments (Figure 6E). Transects were chosen to quantify image signals in regions where bright ARP2/3 signals were either microtubule-associated (Figures 6D,E) or not (Figure 6F). The colocalization was not due to bleed through of the HA into the longer wavelength tubulin channel, because regions within the same image plane analyzed in Figures 6D,E that had bright and saturated ARP2/3 signal had only background tubulin signals at ARP2/3 positive organelles that were not microtubule associated (Figure 6F). Taken together, these results suggest that sub-pools of ARP2/3 and/or ARP2/3-positive organelles associate with microtubules, and there may be instances in which the interaction preferentially occurs with fragmented microtubules and/or microtubule bundles.

In our efforts to identify active ARP2/3, we detected rare instances in which ARP2/3 punctae displayed a more complete overlap with actin. Of the 112 distinct ARP2/3-positive organelles that were analyzed for colocalization, nine displayed a nearly complete signal overlap (Figure 5E). In other instances we detected active pools of ARP2/3 present as clustered ARP2/3 punctae that were superimposed on a broader, but diffuse domain of strong actin signal (Figures 2D, 3A,C). The relatively rare examples of clustered and active ARP2/3 were not counted in the total of 9 puncate that colocalized with actin. Both of above examples could reflect local pools of active ARP2/3 that generated membrane-associated actin meshworks that cannot be resolved in the light microscope. The locations of putatively active ARP2/3 did not correlate with cell shape, and were observed within cell protrusions (Figures 2D, 3A), at the cortex near indentations (Figure 3A), and at different locations within the trans-vacuolar space (Figures 3C, 5B). Although *arp2/3* mutants have a more simple pavement cell shape, this result does not support a simplified scheme in which ARP2/3-generated “fine actin filaments” function constitutively within lobes to drive local cell expansion (Li et al., 2003).

**Figure 7:**
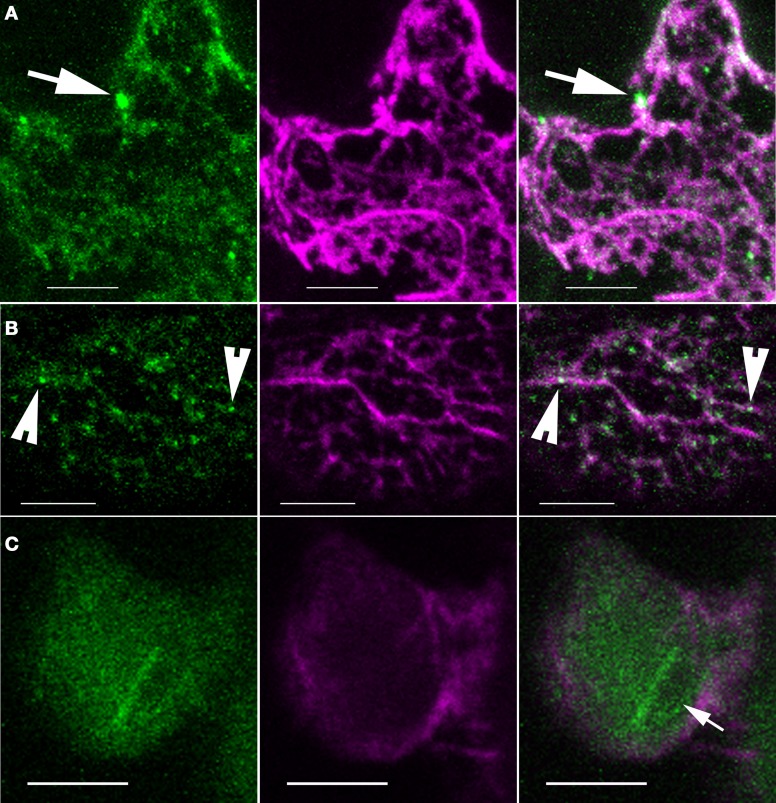
**ARP2/3 localization patterns are not obviously altered in *sra1* pavement cells. (A–C)** Single channel and image overlay projections of confocal images of ARP2/3 (green) and actin (magenta) signals. **(A)** ARP2/3 punctae of differing size classes are detected adjacent to actin-positive structures as indicated by the arrows. Maximum projection of 14 image planes containing 4.6 μm of cell depth. **(B)** Granular ARP2/3 signal and small punctae colocalize with actin bundles as indicated by the arrowheads. **(C)** The ARP2/3-positive perinucleolar bar is present in *sra1* pavement cells. The arrow indicates the nucleolus. Bars = 5 μm.

In a parallel set of experiments, we took a genetic approach to determine if signaling through the W/SRC, which is the sole pathway for ARP2/3 activation (Zhang et al., 2008), is required for the organelle association of ARP2/3 or its association with actin bundles. We crossed the ARPC4:HA marker into *sra1*, a mutant background in which W/SRC signaling to ARP2/3 is severely compromised (Basu et al., 2004; Brembu et al., 2004). The power of this approach was limited by the fact that the actin cytoskeleton tended to be poorly preserved in *sra1* (Figure 7). In general, the actin bundles had a more diffuse appearance and there were fewer isolated filaments. This is likely a limitation of chemical fixation, because live cell-imaging of the F-actin in other W/SRC and ARP2/3 mutant backgrounds failed to reveal such obvious differences (Mathur et al., 2003a; Djakovic et al., 2006). This technical issue precluded quantitative comparisons between genotypes; however, it was possible to qualitatively compare ARP2/3 localization in *sra1* with the wild type. A nuclear pool of ARP2/3 was frequently detected in *sra1* pavement cells, and the bright intranuclear ARP2/3 rods were also present (Figure 7C). Likewise, bright ARP2/3-positive organelles of different sizes were also present in *sra1* (Figures 7B,C), and as observed for the wild type, the vast majority of the bright ARP2/3 organelles in *sra1* were in apparent contact with actin bundles (Figures 7A,B).

## Discussion

We report on the existence of at least five different intracellular pools of ARP2/3 in pavement cells. In addition to a small soluble cytosolic pool (Figure 1A), we detected two biochemically distinct populations of membrane-associated ARP2/3 on sucrose density velocity gradients (Figure 1B). The localization of ARP2/3 to organelles with diverse morphologies and differing abundances are also consistent with the existence of multiple organelle surfaces that accumulate ARP2/3 (Figures 2, 3, 5). We also detected a diffuse intranuclear pool of the complex, and a distinct sub-nuclear pool that assembled into an unusual rod-like structure adjacent to the nucleolus (Figure 4). These results are consistent with metazoan ARP2/3 systems that include nuclear functions and actin polymerization at multiple organelle surfaces (Welch et al., 1997; Strasser et al., 2004; Rottner et al., 2010). The plant model, therefore, is likely to provide broadly useful data on the cellular control of ARP2/3.

### Plant ARP2/3: abundant, concentrated at multiple locations, and mostly inactive

In non-plant systems ARP2/3 is clustered at sites of activation, and it colocalizes with dense populations of actin filaments that are not resolved in the light microscope. In leaf pavement cells we detected rare events in which organelle clusters or individual ARP2/3-positive organelles colocalized with actin (Figures 3A,C). This putatively active pool may promote organelle fusion events (Eitzen et al., 2002) or may locally tubulate membrane to promote cargo sorting and vesicle trafficking from a specific endomembrane compartment (Kaksonen et al., 2003; Derivery et al., 2009; Gomez and Billadeau, 2009). At a cellular scale, the locations of active ARP2/3 were not restricted to lobes, as would be expected if cell protrusions defined locations of persistent polarized growth that required ARP2/3 activity (Li et al., 2003). Three-way cell wall junctions are potential sites for ARP2/3 activation based on the clustering of BRK1 and SCAR1 at that location (Dyachok et al., 2008). Although certainly present at 3-way cell wall junctions in some instances, we detected broadly distributed ARP2/3-positive organelles that did not display any noticeable pattern as a function of cell shape or neighboring cell walls. We do not think the low level of colocalization between ARP2/3 and actin is merely an artifact of chemical fixation, because our methods efficiently preserved ARP2/3 and actin signals. However, the detection of transient, organelle-associated actin is notoriously difficult (Eitzen et al., 2002; Fehrenbacher et al., 2004), and we may not detect all of the active ARP2/3 in the cell.

We report that the vast majority of ARP2/3-positive organelles are not densely coated with actin, and instead these unknown organelles tended to be adjacent to thick actin bundles. This type of actin association did not require ARP2/3 activation, because when W/SRC activation was disrupted in the *sra1* background, both the organelle association of ARP2/3 and its association with actin bundles were not noticeably affected (Figure 7). The positioning of the ARP2/3-positive organelles adjacent to actin bundles is really not consistent with a mechanism of ARP2/3-dependent rocketing motility. A similar actin-associated pool of ARP2/3 was reported in tobacco BY-2 cells, and was interpreted to reflect the output of active ARP2/3 (Maisch et al., 2009). However, this largely non-overlapping pattern is not what would be expected if ARP2/3 was activated and incorporated into an actin meshwork. Furthermore, the ~μm scale ARP2/3 punctae are resolved in the light microscope and are orders of magnitude larger than individual complexes that would generate branched actin filaments.

Our suggestion that ARP2/3 exists in a large latent pool is consistent with several other previous observations. Arabidopsis ARP2/3 is moderately abundant, roughly at a 1:5 molar ratio to ACTIN (Kotchoni et al., 2009). Based on actin filament elongation rates, the cellular concentration of G-actin is estimated to be ~50 μM (Staiger et al., 2009). If ARP2/3 is ~10 μM in cells, there would be on the order of ~10^4^ ARP2/3 complexes per μm^3^ of cytosol or on the surface of a 1 μm-diameter organelle that is saturated with ~70 nm diameter ARP2/3 complexes (Robinson et al., 2001). If fully active, these pools of ARP2/3 would generate a dense gel-like actin network and obvious bright halos of organelle-associated actin. This is clearly not the case; both immunolocalization and live cell imaging experiments depict actin cytoskeleton in pavement cells is relatively sparse and filamentous (Qiu et al., 2002; Mathur et al., 2003a; Djakovic et al., 2006; Smertenko et al., 2010). The barely noticeable actin phenotypes in pavement cells (Mathur et al., 2003a; Djakovic et al., 2006) and the subtle, 10% reduction in F-actin signal in distorted mutant trichomes compared to wild-type (El-Assal et al., 2004a) are also consistent with the existence of large inactive pools of ARP2/3 in the cell. The major challenge now is to determine which organelle systems participate in ARP2/3 activation and its cellular function.

Our cell fractionation and sucrose velocity data do not identify the ARP2/3 positive organelles, but they do provide some useful clues about the cellular control of ARP2/3 activation in plant cells. Here we show that both Arabidopsis ARP2/3 and the W/SRC complex subunit NAP1 bind strongly and specifically to membranes (Figures 1, 2). A large pool of the upstream ROP activator SPK1 (Basu et al., 2008) and the W/SRC subunit NAP1 (Zhang et al., 2013), clearly colocalize with the ER. The most complete overlap of dense ARP2/3 microsomes with NAP1 with SEC12-positive membranes suggests a possible interaction between ARP2/3 and specialized domains of the ER (Figure 1B). However, the association may be weak, because unlike NAP1 (Zhang et al., 2013), obvious ER-like localization patterns were not consistently observed. ARP2/3 may accumulate at different organelle surfaces, because unlike SCAR2 and NAP1, ARP2/3 had a bimodal distribution on sucrose velocity gradients (Figure 1B). The less dense ARP2/3 membranes may not correspond to the ER, because they did not overlap with PDI, another ER marker protein that was present in low density microsomes (Figure 1B). These crude organelle separation assays, and the localization of ARP2/3 to organelles of diverse sizes and shapes, suggest that multiple organelle systems may participate in ARP2/3-regulation. ARP2/3 may accumulate on the surface of different types of organelles, a single type of organelle at different stages of biogenesis, and/or a membrane subdomain within a larger organelle surface.

### Functional implications for coupling ARP2/3 to the actin and microtubule systems

We believe that the moderate level of colocalization of ARP2/3 and actin [PCC values of 0.61 ± 0.17 (*N* = 48)] largely reflects ARP2/3-positive organelles that are efficiently coupled to the actin network, but not necessarily active. The simplest and most likely explanation is that ARP2/3 is cargo on organelles that undergo acto-myosin-dependent transport. Myosin mutants have distorted-like trichomes and pavement cell shape defects that are consistent with an involvement of motors in cell shape control in the epidermis (Prokhnevsky et al., 2008; Ojangu et al., 2012). Further work is needed to determine if organelle-associated ARP2/3 has a direct role in either coupling organelles to the actin bundle network or controlling other aspects of subsequent myosin-dependent transport. Pavement cell strain rates are slow: only ~1%/h. Perhaps growth is pulsatile and there are sporadic events that require localized ARP2/3-dependent filament nucleation. In this scenario, regulated acto-myosin transport of latent ARP2/3 could be part of a complicated cellular mechanism to initiate and reinforce localized ARP2/3 activation.

We also detected organelle-associated ARP2/3 that displayed unexpected associations with microtubules (Figure 6). About 50% of the >0.5 μm class ARP2/3 punctae were either adjacent to or partially overlapped with the anti-tubulin signal (Figure 6), and the overall PCC of ARP2/3 and microtubules was not significantly different than that of ARP2/3 and actin. Thirty percent of the colocalizing particles had a more complete overlap with the microtubule and were observed at putative microtubule fragments or at microtubule ends. Additional work is needed to determine what might mediate a physical interaction between ARP2/3 and microtubules and the biochemical consequence of this interaction, but at the moment, our limited data are consistent with a hypothesis in which a sub-pool of ARP2/3 locally destabilizes microtubules. The proposed functional antagonism between ARP2/3 and microtubules would be consistent with a known genetic interaction in which SCAR2 appears to destabilize the cellular organization of microtubules in trichomes (Zhang et al., 2005), and the lack of this activity in the distorted mutants may explain their twisted growth habit (Buschmann et al., 2009).

### ARP2/3: A potential importance of a nuclear pool

We also consistently detected an intranuclear pool of ARP2/3 in pavement cells (Figure 4). Although reports of endogenous nuclear ARP2/3 are rare, our results are consistent with previous reports that other plant ARP2/3 pathway components and actin have been detected in the nucleus (Cruz and Moreno Diaz de la Espina, 2008; Zhang et al., 2010; Dyachok et al., 2011). Historically in the actin field, the topic of F-actin in the nucleus in general has been controversial, and the precise function of nuclear ARP2/3 is not known (Goley et al., 2006; Yoo et al., 2007). However, there is an increasing recognition that nuclear pools of nucleation promoting factors and ARP2/3 are likely to have a functional importance (Weston et al., 2012). We also not only detected nuclear ARP2/3, but a remarkable perinucleolar ARP2/3 rod that was oriented toward cytoplasmic actin bundles that were positioned near the outer nuclear envelope (Figure 4). Given that these ARP2/3 rods are present in *sra1*, their assembly does not require known signal transduction and activation through W/SRC. Given its position between the nucleolus and cytoplasmic actin bundles, the perinucleolar ARP2/3 rod may mediate efficient coupling of ribosome export or recycling with the extranuclear actin bundle network. Alternatively, the rod may function as a mechanical gene-expression sensor that links a nucleolar domain and with the cytoplasmic actin bundle system. There is so much interesting biology centered on plant ARP2/3 that has yet to be discovered.

## Conclusion

In Arabidopsis, cell types differ greatly in their threshold requirements for SCAR-dependent activation of ARP2/3 (Zhang et al., 2008). In trichomes, the apical plasma membrane of stage 4 cells may be one location of active ARP2/3 (Dyachok et al., 2008). Our accounting of ARP2/3 localization in pavement cells reveals at least 5 intracellular pools, most of which appear to be organelle-associated and inactive. ARP2/3-positive organelles may relocalize using the acto-myosin system. It remains to be determined how this localization pattern relates to the commonly observed branched filament nucleation that originates from actin bundles (Staiger et al., 2009) and the ability of ARP2/3 to influence actin and microtubule networks at whole cell spatial scales(Szymanski et al., 1999; Le et al., 2003). It may be that localized ARP2/3-activation, actin bundle network reorganization, and regulated organelle positioning are interdependent processes that feed back on one another to bias the patterns of organelle transport, secretion, and growth. Our biochemical and cytological measurements depict the cellular pool of ARP2/3 as mostly inactive with respect to actin filament nucleation, but potentially active in other ways, such as promoting the coupling of specific organelles with the cytoskeleton, affecting the local behavior of microtubules, or providing some form of physical continuity between the intranuclear and cytoplasmic space. The known upstream regulators of ARP2/3, such as the DOCK-family GEF SPK1, and the W/SRC subunits NAP1 and SCAR2 are present on the ER surface (Zhang et al., 2010, 2013), but ARP2/3 accumulates on a heterogeneous population of organelles, many of which bear no resemblance to a tubulated ER network. It therefore appears likely that the cellular control of the SPK1-W/SRC-ARP2/3 pathway involves multiple organelle systems and probably multiple layers of regulation that go beyond the simplistic idea that ROP/Rac-binding is sufficient to fully activate the pathway. Additional double- and triple-labeling experiments are needed to define exactly where and when ARP2/3 is active. Time-lapse imaging of GFP-tagged ARP2/3, combined with new image analysis methods (Zhang et al., 2011), and the collection of distorted mutants provide realistic opportunities to systematically peel away the layers of ARP2/3 activation and discover its relationship to cell shape control.

## Methods and materials

### Strains and growth conditions

Arabidopsis ecotype Col-0 was used as wild-type. An Arabidopsis transgenic line expressing the genomic content of ARPC4:HA in an *arpc4-t1* background was described as published (Kotchoni et al., 2009). Arabidopsis seedlings for immunostaining and microsomal fraction preparation were grown on 1/2 MS media supplemented with 1% sucrose and 0.8% Bacto agar (BD, Franklin lakes, NJ) in a 22°C growth chamber under continuous light. *pir-3* allele (Basu et al., 2004) was used to cross with ARPC4:HA;*arpc4-t1* to create *sra1*;ARPC4HA;arpc4-t1 allele by selecting distorted mutants that are resistant to basta and homozygous for *arpc4-t1* T-DNA insertion in the F2 population.

### Cell fractionation and western blotting

For the cell fractionation experiments, protein fractions were isolated from 25 DAG plate-grown ARPC4:HA; *arpc4* plants (24 h light) using a homogenization buffer containing 20 mM Hepes/KCl pH 7.2, 50 mM KOAc, 2 mM Mg(OAc)2, 250 mM Sorbitol, 1 mM EDTA, 1 mM EGTA, 1 mM DTT, 1 mM PMSF and 1% (v/v) protease inhibitor cocktail (Sigma, St. Louis, Missouri). All steps were performed in wet ice except as noted otherwise. Two grams of shoots were homogenized in 10 ml homogenization buffer on ice, and then filtered through pre-wetted double layers of Miracloth to remove the cell debris. A small portion of the flow through was saved as an equal proportion of total protein. Then the remainder was spun at 1000 g for 10 min at 4°C in a Beckman F0850 rotor. The supernatant was then split: part was centrifuged at 200,000 g for 1 h at 4°C in a Beckman Optima MAX tabletop ultracentrifuge and part was centrifuged at 10,000 g for 1 h in a Beckman Avanti 30 centrifuge, F0850 rotor. The 10,000 g supernatant was then centrifuged at 200,000 g for 1 h at 4°C in the Beckman ultracentrifuge. Fractions were loaded on SDS-PAGE gels with cell fractions loaded in equal proportion, and blotted against primary antibodies of anti-NAP1 (1:1000), anti-SCAR2 (1:1000), anti-ARP3 (1:870), and anti-Pep carboxylase (1:5000, Rockland Immunochemicals, Gilbertsville, PA).

Proteins were separated on SDS-PAGE gels using a BioRad Mini-Protein3 Cell (Hercules, CA) and transferred to 0.2 μm reinforced nitrocellulose membranes (Whatman Optitran BA-S 83, Dassel, Germany) in chilled 25 mM Tris, 192 mM glycine, 20% (v/v) methanol, 0.1% SDS buffer using a Biorad Mini Trans-Blot Electrophoretic transfer cell (Hercules, CA) for 2 h at 80 V. Membranes were rinsed briefly with TTBS and then blocked in 5% dry milk in TTBS (50 mM TrisHCl, 100 mM NaCl and 0.1% Tween-20, PH 7.5) for 1 h at room temperature. Primary antibodies were incubated overnight at 4°C, washed 3 (for anti-NAP, anti-ARP3, and anti-PEP-C) or 4 (for anti-SCAR2) times 10 min at room temperature with chilled TTBS, and then incubated with an HRP-conjugated goat-anti-rabbit secondary antibody (Pierce, Rockford, IL) at 1:50,000 for 1–2 h at room temperature. Signal was detected on x-ray film with SuperSignal West Pico Chemiluminescent Substrate (Pierce, Rockford, IL). The anti-HA antibody was used at 1:250 (MMS-101R, Covance Research Products, Berkeley, CA). No Tween-20 was used in the western blot procedure with anti-HA. Secondary antibody was goat anti-mouse IgG (1:5000, Pierce, Rockford, IL).

### Sucrose velocity gradient separations

Sucrose density gradients in the range of 20–50% (w/v) were prepared in 5-ml tubes using a Gradient Master™ 108 (BioComp Instruments, Inc., Fredericton, NB Canada) following the operator's manual. Light (20%) and heavy (50%) sucrose solutions were prepared in the following buffer: 20 mM HEPES/KOH, pH 7.8, 2 mM EDTA, 2 mM EGTA, 1 mM DTT, 5% glycerol. Tubes were marked for half-full by placing in the Marker Block as provided by the manufacturer and filled with light sucrose solution. The heavy sucrose solution was then layered from the bottom until the heavy-light interface rose precisely to the half-full mark. Gradients were prepared by selecting SW50 rotor and the short 2-step process for the 20–50% (w/v) sucrose concentration from the lists of programs in the Gradient Maker v5.0. The microsomal fraction (200 k pellet) was re-suspended in resuspension buffer (10 mM HEPES/KOH, pH 7.2, 150 mM NaCl, 1 mM EDTA, 10% glycerol, 2 mM DTT, 1% (v/v) protease inhibitors, 1 mM PMSF) and 200 μl of the suspended solution was layered on the top of each gradient tube. Organelles were separated by centrifugation for 28 h (20–50%) at 31,000 rpm (100,000 RCF max) using the MLS50 swinging bucket rotor in the Optima Max Preparative Ultracentrifuge (Beckman Coulter, Inc., Fulerton, CA). After centrifugation, each gradient was divided into 24 fractions by collecting 200 μl per fraction from the top using a Pipetman and later used for western blot analysis.

### Etiolated hypocotyl assay in different genotypes

Cold-treated seeds were surface sterilized and plated onto 1/2 × MS plates, 0.5 cm apart. Plates were put into a 22°C continuous light growth chamber horizontally for 16 h. They were then wrapped in double layers of alumni foil and placed vertically in the same growth chamber. At 7 days after germination, the etiolated hypocotyls were measured or photographed.

### Immunolabeling of HA-tagged ARPC4 and actin by freeze shattering and confocal microscopy

Shoots of Arabidopsis 12 day old seedlings were dissected from the roots using a razor blade and fixed in PEM buffer (100 mM PIPES, 4 mM MgCl_2_, 10 mM EGTA, pH 6.9) containing 400 μM MBS (3-Maleimidobenzoic acid *N*-hydroxysuccinimide ester) and 0.05% Triton X-100 for 20 min at room temperature. The samples were then transferred to PEM buffer containing 200 μM MBS, 0.05% Triton X-100, 2% formaldehyde freshly prepared from paraformaldehyde, and 0.5% glutaraldehyde and for 1 h at room temperature. After the fixation step, the samples were washed twice with PEM buffer containing 0.05% Triton X-100, 10 min for each wash. The samples were then briefly dried with 3 MM Whatman filter paper and lined up on a glass slide. Another glass slide was put on the sample and the glass slide sandwich was frozen in liquid nitrogen. The frozen tissues were shattered with two cold blocks and the shattered samples were transferred to a 6 well dish containing permeabilization buffer [1 × PBS buffer (PH7.4) with 0.1% Triton X-100]. The samples were kept in permeabilization buffer for 1 h, shaking at ~60 rpm. Then the samples were transferred to 1.5 ml eppendorf tubes and the permeabilization buffer was removed using a 1 ml syringe with a needle. The samples were washed with PBST-G buffer (1 × PBS, 0.05% Triton X-100 and 50 mM Glycine) for two times, 5 min each wash. Two hundred microliters of PBST-G buffer was added to each tube and primary antibodies were added at corresponding dilutions. The samples were then kept at 4°C overnight. The next day, primary antibodies were removed with a 1 ml syringe with a needle and the samples were washed with PBST-G 3 × 10 min. Then the secondary antibodies were added to the samples and kept at 4°C overnight. The samples were then washed twice in PBST-G and once in 1 × PBS, with 10 min for each wash. The samples were then mounted on glass slides and examined under confocal microscopy within 2 days. The mouse monoclonal IgM anti-actin (JLA20) antibody (Calbiochem, San Diego, CA) was used at 1:400 dilution. Anti-HA high affinity rat monoclonal antibody (clone 3F10) was used at 1:200 dilution in immunostaining (Roche Applied Science, Indianapolis, IN). Alexa 488 conjugated goat anti-rat secondary antibody was used at 1:200 dilution (Life Technologies, Carlsbad, CA). Rhodamine conjugated goat anti-mouse IgM secondary antibody was used at 1: 400 dilution (Life Technologies, Carlsbad, CA). The immunolabeled samples were examined under a Bio-Rad Radiance 2100 Confocal Laser Scanning Microscope mounted on a Nikon E800 stand. A 40× Plan Apo 1.3 NA oil objective was used in the Radiance 2100 system. A Yokogawa Spinning Disk confocal mounted on an inverted Zeiss Observer.Z1 stand and a 100× Plan Apo 1.46 NA objective were also used. A 488 nm laser line and a 500–560 nm emission filter were used for imaging in the green channel and a 543 nm laser line and a 555–625 nm emission filter (Radiance 2100) or a 561 nm laser line and a 580–653 nm (spinning disk) emission filter were used for imaging in the red channel. All double-labeled images were acquired using sequential laser excitation methods.

### Cell shape quantification and gap measurement

12-DAG soil-grown plants of different genotypes were used for pavement cell shape quantification and gap frequency measurement. Five fully expanded cotyledons were dissected from 5 individual seedlings and stained in 1 μM FM1-43 for 30 min. One to three images were taken from non-overlapping regions in the apical third of each cotyledon using a MRC Bio-Rad 2100 laser scanning confocal microscope with a Nikon 20×/0.75-NA objective. The cell sampling strategy is similar to Zhang et al. (2008). In brief, the confocal image stacks were projected and the cells that intersected the diagonal line were traced with the free-hand tool and measured using ImageJ. To estimate the numbers of skeleton ends, we generated a binary image from the traced pavement cell, and then we generated skeleton ends images after two rounds of Erode. The numbers of ends were counted manually. To count the frequency of gaps in different genotypes, we generated a maximum Z-projection image from each confocal stack. Then we counted the numbers of black holes that were not stained with FM1-43, which indicates the presence of gaps. To analyze the difference of gaps frequency in different genotypes, we calculated the numbers of gaps per mm^2^ based on the size of image field and the numbers of gaps in the field. To compare the cell shape in different genotypes, the one-way ANOVA test was performed using MiniTab software for all parameters.

### Pearson correlation coefficient and particle size quantification

To quantify the colocalization between ARPC4 and ACTIN, confocal image z-stacks that contained ARPC4 and ACTIN in two separate channels were used. PCC was calculated from individual image planes in all cases. For 2-channel images substacks were created from regions of interest in the cell, and a synchronized free hand drawing tool was used to select the maximal region that was within the cell boundary and well-labeled in both channels. The Coste algorithm in coloc 2 colocalization analysis tool in Fiji (http://fiji.sc/) was used to automate image thresholds based on signal intensity distribution and to quantify the Pearson correlation coefficient between the two channels in the selected region. The PSF (Point Spread Function) value of 3.0 and a Coste randomization iteration value of 10 were used. The Pearson's *R*-value above threshold was used for each analysis. The same procedure was applied to quantify colocalization between ARPC4 and tubulin. Confocal image stacks that contained punctate ARPC4 localization patterns were selected and used for ARPC4 size quantification. The image stacks were manually inspected, image planes that contained ARPC4 punctae were selected, and maximum Z-projections of these image planes were created using ImageJ software. The resulting images were thresholded to eliminate background fluorescence and binary images were created. Regions of interest (ROI) were created to include the punctae and the descriptive parameters of the punctae were measured using the Analyze Particles function of ImageJ. Small particles that were less than 4 pixels in size were treated as noise and discarded from quantification list.

### Conflict of interest statement

The authors declare that the research was conducted in the absence of any commercial or financial relationships that could be construed as a potential conflict of interest.
